# Genotyping *Plasmodium vivax* isolates from the 2011 outbreak in Greece

**DOI:** 10.1186/1475-2875-12-463

**Published:** 2013-12-27

**Authors:** Gregory Spanakos, Michael Alifrangis, Mette L Schousboe, Eleni Patsoula, Nicholas Tegos, Helle H Hansson, Ib C Bygbjerg, Nicholas C Vakalis, Maria Tseroni, Jenny Kremastinou, Christos Hadjichristodoulou

**Affiliations:** 1 Hellenic Centre for Diseases Control and Prevention, Marousi, Greece; 2 Department of Parasitology, Entomology and Tropical Diseases, National School of Public Health, Athens, Greece; 3 Centre for Medical Parasitology, Department of International Health, Immunology and Microbiology, University of Copenhagen, Copenhagen, Denmark; 4 Department of Infectious Disease, Copenhagen University Hospital, Copenhagen, Denmark; 5 Department of Hygiene and Epidemiology, Medical Faculty, University of Thessaly, Thessaly, Greece

**Keywords:** Outbreak, Greece, *Plasmodium vivax*, Genotyping, Merozoite surface protein-3α, Microsatellites

## Abstract

**Background:**

*Plasmodium vivax* malaria was common in Greece until the 1950s with epidemics involving thousands of cases every year. Greece was declared free of malaria by the World Health Organization in 1974. From 1974 to 2010, an average of 39 cases per year were reported, which were mainly imported. However, in 2009 and 2010 six and one autochthonous cases were reported culminating with a total of 40 autochthonous cases reported in 2011, of which 34 originated from a single region: Laconia of Southern Peloponnese. In this study the genotypic complexity of the *P. vivax* infections from the outbreak in Greece during 2011 is described, to elucidate the possible origin and spread of the disease.

**Methods:**

Three polymorphic markers of *P. vivax* were used*; Pvmsp-3α* and the microsatellites m1501 and m3502 on *P. vivax* isolates sampled from individuals diagnosed in Greece. Thirty-nine isolates were available for this study (20 autochthonous and 19 imported), mostly from Evrotas municipality in Laconia region, in southern Greece, (n = 29), with the remaining representing sporadic cases originating from other areas of Greece.

**Results:**

Genotyping the Evrotas samples revealed seven different haplotypes where the majority of the *P. vivax* infections expressed two particular *Pvmsp-3α*-m1501-m3502 haplotypes, A10-128-151 (n = 14) and A10-121-142 (n = 7). These haplotypes appeared throughout the period in autochthonous and imported cases, indicating continuous transmission. In contrast, the *P. vivax* autochthonous cases from other parts of Greece were largely comprised of unique haplotypes, indicating limited transmission in these other areas.

**Conclusions:**

The results indicate that several *P. vivax* strains were imported into various areas of Greece in 2011, thereby increasing the risk of re-introduction of malaria. In the region of Evrotas ongoing transmission occurred exemplifying that further control measures are urgently needed in this region of southern Europe. In circumstances where medical or travel history is scarce, methods of molecular epidemiology may prove highly useful for the correct classification of the cases.

## Background

Annually, an estimated 219 million people are infected with malaria and the disease is endemic in 104 countries [1]. *Plasmodium falciparum* malaria is associated with the highest mortality and is accordingly prioritized in research and control. Another species, *Plasmodium vivax*, has gained less scientific attention despite being the most widely distributed malaria species endemic in tropical and subtropical countries worldwide, with an estimated 2.8 billion people currently at risk [2-4]. It is estimated that at least 130 million people are infected with *P. vivax* annually [5], causing significant economic and financial burden to affected countries [6].

In historic Europe, *P. vivax* malaria was endemic in many countries, reaching as far as Finland and England in the north [6,7]. Malaria disappeared from Europe in the mid-20th Century, likely as a result of a combination of various factors, including improved housing conditions, better health care services, and the implementation of various malaria eradication programmes [8,9].

According to the World Malaria report in 2011, “The European Region has a real possibility of becoming the first to achieve the complete elimination of malaria within the next few years, and aims to do so by 2015” [10]. Within the European Union (EU), all EU Member States are considered malaria-free [11]. The likelihood of re-emergence of autochthonous *P. vivax* malaria in southern Europe due to factors such as global warming, and even the ongoing economic crisis, has been debated. Various studies indicate an increased risk imposed by the presence of suitable vectors [12] and the increased number of travellers and immigrants from endemic countries [13,14].

In Greece, *P. vivax* malaria was a common disease before the Second World War, with epidemics involving thousands of cases every year. Following a national eradication programme between 1946 and 1960, Greece was declared free from malaria by the World Health Organization in 1974. Malaria is a mandatory notifiable disease in Greece. Accordingly, from 1974 to 2010, an average of 39 cases per year were reported, most of which were imported [15,16] presumably due to the large number of immigrants (estimated to be close to one million) that occasionally or permanently live in Greece [17], or due to travellers returning from malaria-endemic countries. However, 17 sporadic autochthonous *P. vivax* cases were detected during the years 1991, 1999, 2000, 2009, and 2010 [15,16]. In Greece, the potential for re-emergence of malaria transmission is present due to the widespread occurrence of several anopheline vector species. *Anopheles sacharovi, Anopheles maculipennis, Anopheles superpictus* and *Anopheles claviger* are among the species identified in the country [18,19]. *An. sacharovi* was implicated as the vector species responsible for transmission of a majority of the malaria cases in Greece prior to the nationwide eradication in 1974 [20].

In 2011, a total of 40 autochthonous *P. vivax* cases were reported, 34 of which were derived from a single region, Evrotas municipality in Southern Peloponnese [16]. This outbreak in Evrotas calls for concern and should be explored further. One important aspect to investigate is the epidemiological pattern of occurrence and spread of autochthonous *P. vivax* malaria in the affected areas. With this knowledge, it may be possible to determine at-risk populations and identify possible areas to focus interventions, which is crucial if similar *P. vivax* epidemics emerge in the future. The use of molecular tools that genetically fingerprint *P. vivax* parasites could provide a powerful tool as an adjunct to more classical epidemiological investigations.

Many molecular markers have been used to genotype *P. vivax*. They can be classified into two categories, those that are under natural selection and those that are evolutionarily neutral or nearly neutral. *Plasmodium vivax* circumsporozoite protein (*Pvcs*), merozoite surface protein-1 (*Pvmsp-1*), and merozoite surface protein-3α (*Pvmsp-3α*) are genes that have been widely used for genotyping, as these are highly polymorphic and under natural selection [21].

Recently, microsatellite markers (MS) have increasingly been used in studies of *P. vivax* diversity. They are less laborious to perform, considered selectively neutral, and often increase the resolution compared to genetic markers such as *Pvcs*, *Pvmsp*-1 and *Pvmsp-3α*[22-26]. Three molecular markers were used (*Pvmsp-3α* and the MS m1501 and m3502) to genotype *P. vivax* isolates sampled from the majority of infected individuals during 2011 in Greece. With this approach it is most likely possible to substantiate whether the outbreak was caused by multiple *P. vivax* re-introductions, and enables identification of areas with continuous transmission. These areas likely present locations of higher transmissibility of malaria, identification of which can aid in enhancing and expanding the findings of classical epidemiological investigations. Recording the genotypes involved in the 2011 outbreak is considered useful for future comparisons and will help guide implementation of precautionary control measures for potential future outbreaks of *P. vivax* malaria in Greece.

## Methods

### Sample collection and DNA extraction

Blood samples were sent to the Greek Malaria Reference laboratory in Athens by physicians from hospitals across the country or by the Hellenic Centre for Disease Control and Prevention (HCDCP). Parasitic DNA was isolated from 0.5 ml of peripheral blood, using either the QIAamp DNA mini kit (QIAGEN GmbH, Hilden, Germany) or the Magcore automated system (RBC Bioscience, New Taipei City, Taiwan). For the QIAamp DNA mini kit, 1.5 ml TE buffer was added to the blood sample and the suspension was centrifuged for 1 min at 5,000 xg. The pellet was resuspended in 180 μl of PBS. Subsequent procedure was carried out according to the manufacturer’s recommendations and the DNA was finally diluted in 100 μl of AE buffer. For the Magcore the isolation was implemented using 500 μl of blood according to the recommendations of the manufacturer.

### PCR amplification of the *Pvmsp-3α* gene and restriction fragment length polymorphism analysis

The presence of *P. vivax* parasites in the samples was confirmed using a previously described PCR-based method [27].

PCR amplification of a region of the *Pvmsp-3α* gene was performed as previously described [28]. Five μl of PCR product were digested with the restriction endonucleases HhaI and AluI to separate different genotypes, using buffers provided by the manufacturer (New England Biolabs, Ipswich, MA, USA). The resulting digests were run by electrophoresis on a 2% agarose gel and the bands were visualized on a UV plate. The amplicons were classified according to size as A (~1,900 bp), B (~1,500 bp), or C (~1,150 bp). A number was used to characterize the specific RFLP pattern.

### Sequencing of fragments of the *Pvmsp-3α* gene

Fourteen of the positive samples were randomly selected for sequencing of fragments of the *Pvmsp-3α* gene to confirm the results obtained from RFLP. Outer PCR products were amplified in 100 μl reactions. The total volume was electrophoresed on a 2% agarose gel and the band was excised over a UV plate. DNA from the band was isolated using the QIAquick gel extraction kit (QIAGEN GmbH), according to the manufacturer’s instructions. Sequencing was performed by the Department of Immunology and Histocompatibility, School of Medicine, University of Thessaly (Larissa, Greece), using the primers in Table 1.

**Table 1:** The primers used for sequencing of the MSP-3α PCR products

**Primer name**	**Sequence**
Pv-N1	5′-GACCAGTGTGATACCATTAACC-3′
Pv-N2	5′-ATACTGGTTCTTCGTCTTCAGG-3′
N4	5′-CCGTGCGTCTTTGCCTCTTCCG-3′
N3adf	5′-GAAGCGGAAATAGCCGTAGAG-3′
N5adf	5′-CCAGAAAGCGAAAGAAGCTG-3′
N7	5′-TAAAGCAAATGTGGAAGCAG-3′
N3bce	5′-AGCAATTGAAGTAGCAAAGG-3′
N6adf	5′-TTTCTCAGCATTGGTTYCG-3′
N4adf	5′-CCACTTCGGCAGCTATTTCTG-3′
N5	5′-CCAGAAAGCGAAAGAAGCTG-3′

Primers designated as adf were only used for type A amplicons sequencing. The primer N3bce was used only for type B and C amplicons, while primer N5 was used only for sequencing of the type B amplicons.

### Amplification and fragment analysis of the *Plasmodium vivax* samples using the microsatellites (MS) m1501 and m3502

The two MS, m1501 and m3502, were amplified by a semi-nested PCR and analysed on an ABI 3730XL Genetic Analyzer (Applied Biosystems, Foster City, California, USA) using primers and protocol described by others [26]. The length of the individual MS alleles was determined by reference to the Genescan 500 Liz size standard (Applied Biosystems) using Genemapper vs. 4.1 (Applied Biosystems). Whenever a sample was negative at one or both of the loci, repetition of PCR was performed with 2 μl DNA template (instead of 1 μl) in the primary PCR.

This work has been approved by the HCDCP and all samples were anonymous.

## Results

### Origin of the *Plasmodium vivax* samples

**Figure 1:**
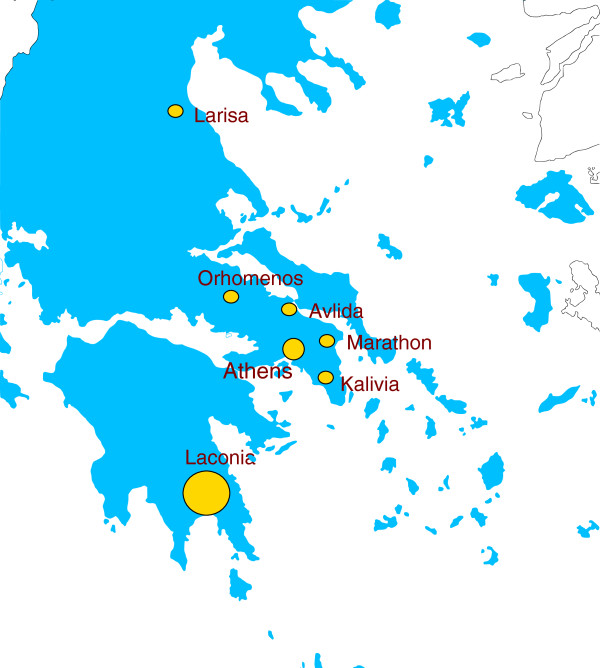
**Map of Greece indicating the locations of the cases included in the study.** The size of the dot is roughly proportional to the number of the samples.

One *P. vivax* isolate from a Greek citizen infected during 2009 and 38 *P. vivax* isolates from patients in 2011 were available for this study. They were all classified as imported or autochthonous according to the HCDCP [16]. All imported cases were undocumented immigrants from Southeast Asian countries endemic for malaria and complete travel and medical history could not be obtained for these cases. Most of the 2011 samples available were derived from the region of Laconia, specifically the municipality of Evrotas (n = 28), while sporadic cases were identified in Athens (n = 5), Kalivia (n = 1), Marathon (n = 1), Avlida (n = 1), Orhomenos (n = 1) and Larisa (n = 1) (Figure 1). The *P. vivax* isolates of 2011 were identified during May (n = 1), July (n = 1) August (n = 7), September (n = 22) and October (n = 7).

With regard to the Evrotas outbreak, with the exception of the four cases diagnosed in July that were not included in this study, there was good representation of the cases among the samples studied per month. The *P. vivax* patients diagnosed in May (n = 2), August (n = 7), September (n = 32), and October (n = 10) provided this study with access to one, four, 18 and five blood samples for the respective months. Of these 28 samples, 14 were isolated from imported cases and 14 from autochthonous cases. The one *P. vivax* isolate diagnosed in 2009 came from a Greek citizen residing in Evrotas with no travel history to a malaria-endemic country.

All five *P. vivax*-positive samples from Athens were from imported cases. Four of these came from patients diagnosed in hospitals in Athens and one case was identified during active surveillance activities of the HCDCP. The *P. vivax* samples from Kalivia, Marathon, Avlida, Orhomenos, and Larisa were derived from autochthonous cases.

### The diversity of *Plasmodium vivax* infections sampled in Greece based on *Pvmsp-3α* and microsatellite m1501/m3502 genotyping

**Figure 2:**
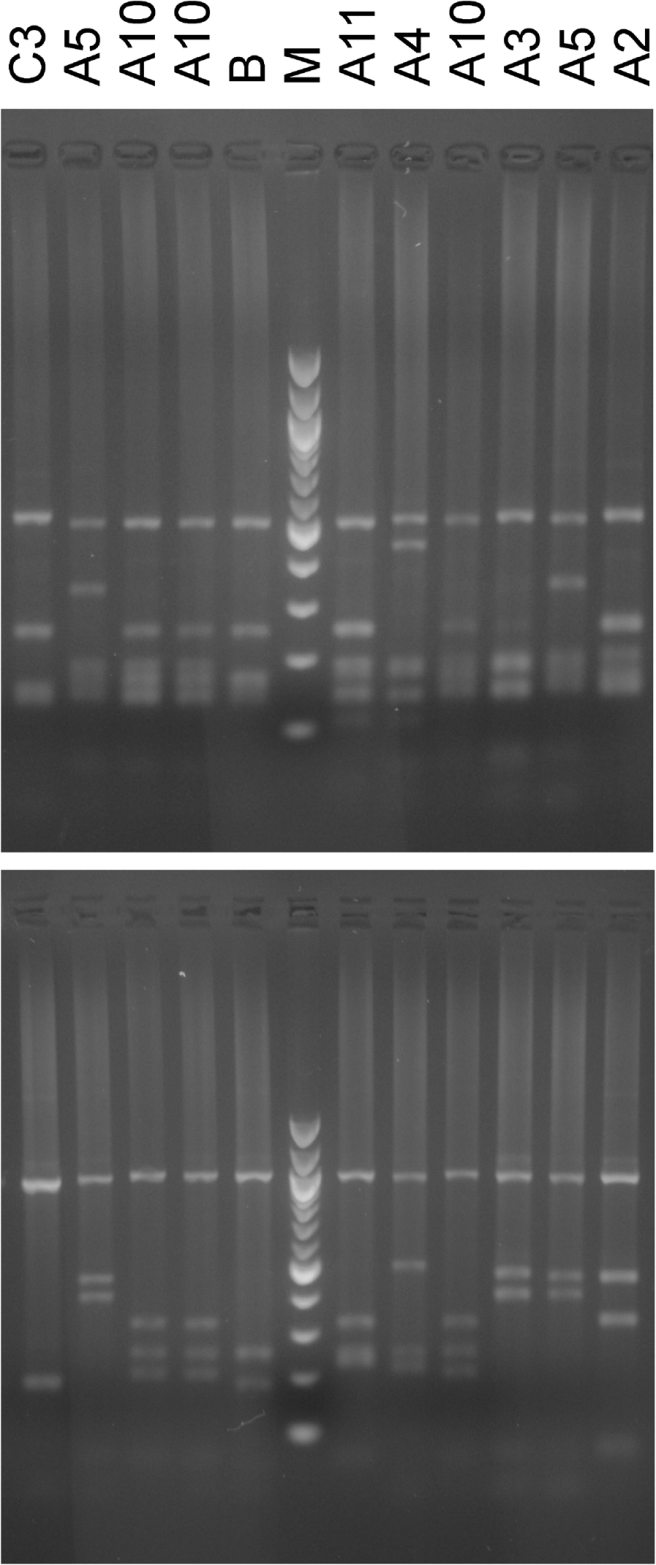
**Representation of the *Pvmsp-3α *results for a subset of *Plasmodium vivax *samples.** The photographs shown are RFLP patterns obtained after digestion of *Pvmsp-3α* products with Alu I (upper photo) and Hha I (lower photo) restriction endonucleases. The characterization of each pattern is indicated at the top.

The genotyping of *Pvmsp-3α* revealed limited diversity with eight different *Pvmsp-3α* RFLP profiles found among 36 of the 39 samples that were *Pvmsp-3α* positive (Figure 2). The A10-genotype comprised the majority of *P. vivax* infections (n = 23), while eight *P. vivax* samples expressed other A-genotypes. Four *P. vivax* infections expressed the C genotype, one *P. vivax* infection expressed the B genotype, and the three remaining samples were consecutively negative by *Pvmsp-3α* PCR (Table 2). Sequencing of the *Pvmsp-3α* amplicons confirmed that the genomic region amplified was as expected. Representative sequences were deposited in EMBL nucleotide sequence Bank with Acc Nos HE961831-4 and HE962511. The ten A10 type genotypes were 1,852 bp in length and were genetically identical. The type A11 sequence was sized 1,876 bp and the type B sequence was sized 1,447 bp. Two sequences of type C, sized 1,093 bp, differed in one nucleotide substitution (G or A) at position 851.

**Table 2:** **The distribution of combined *Pvmsp-3α *** **and microsatellite 1501/3502 haplotypes in *Plasmodium vivax *** **samples collected in Greece**

**Month/year**	**Pvmsp-3α**	**m1501**	**m3502**	**Number of cases**	**Location**
09/2009	A11	107	191	1U	Evrotas
05/2011	A3	150	151	1U	Evrotas
08/2011	A10	128	151	2U	Evrotas
	A10	121	142	1I	Evrotas
	A3	193	142	1I	Evrotas
09/2011	A10	128	151	5U-5I	Evrotas
	A10	121	142	3U-3I	Evrotas
	A10	128	#	1I	Evrotas
	B	262	142	1I	Evrotas
10/2011	A10	128	151	2U	Evrotas
	A10	100	151	1U	Evrotas
	#	286	128	1I	Evrotas
	#	286	#	1I	Evrotas
07/2011	A5	121	151	1U	Avlida
08/2011	C1	276	133	1U	Larisa
	C3	193	142	2U	Kalivia-Marathon
09/2011	A5	135	176	1U	Orhomenos
	A5	207	142	1I	Athens
	C3	125	184	1I	Athens
	A4	100	142	1I	Athens
10/2011	A2	100	151	1I	Athens
	#	286	142	1I	Athens

“U” = autochthonous cases and “I” = imported cases, according to classification from HCDCP [16]. “#” = the genotype could not be determined.

All 39 *P. vivax* samples were positive for the MS m1501 locus, while two samples were negative for the MS m3502. In total, 12 and seven different MS alleles were identified for the m1501 alleles and m3502, respectively. Combining the MS m1501 and m3502 into haplotypes, and omitting the few MS negative samples, revealed a higher diversity with 15 different combinations of MS compared to *Pvmsp-3α*. The majority of *P. vivax* infections expressed the 128–151 haplotype (n = 14) followed by 121–142 (n = 7), 193–142 (n = 3) and 100–151 (n = 2) while 11 *P. vivax* samples expressed unique MS haplotype combinations. Combining MS with the *Pvmsp-3α* showed that all 121–142 and 128–151 (n = 21) were of the A10 genotype (Table 2). Two of the MS 193–142 haplotypes and the MS 125–184 haplotype were of the C3 genotype. The 276–133 MS genotype presented a C3 *Pvmsp-3α* RFLP profile, but as mentioned, sequencing revealed a one base substitution (sequence with Acc No HE961834), compared to the other C3 sequence (HE962511), so it was characterized as C1. Finally 121–151, 207–142 and 135–176 were of the A5 genotype. In three cases, the same MS type 193–142 was of different *Pvmsp-3α* genotypes (A3 and C3) and in two cases, the 100–151 MS type was of different *Pvmsp-3α* genotypes (A2 and A10). Indications of multiclonality in the samples studied were not found.

### The origin and spread of *Plasmodium vivax* infections in Greece 2011 based on *Pvmsp-3α* and microsatellite m1501/m3502 genotyping

#### Evrotas municipality, Laconia region

**Figure 3:**
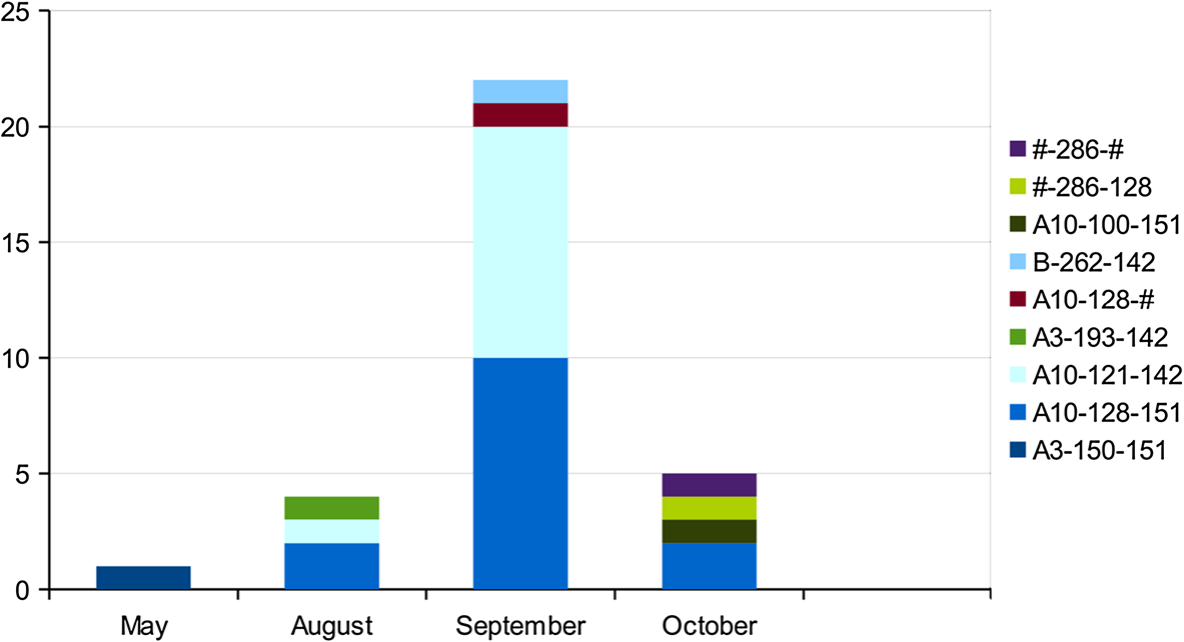
**The combined *Pvmsp-3α*, microsatellite (MS) m1501- m3502 haplotypes derived from the cases in Laconia during 2011.** The number of cases as a function of month of sampling are shown where each *P. vivax* isolate is combined into *Pvmsp-3α* MS m1501-m3502 haplotypes. #: denote unknown genotype at one or more of the markers.

In May of 2011, the first recorded appearance of *P. vivax* infection was documented, where the haplotype A3-150-151 appeared in a Greek child in Evrotas (Table 2). This haplotype did not re-appear throughout the season among the studied samples. Seven out of 27 samples examined until late October expressed the A10-121-142 haplotype, while 14 expressed the A10-128-151 haplotype, which was predominant by late September (see Figure 3). These haplotypes were present almost equally in imported and autochthonous cases (see Table 2). Two imported cases expressed unique haplotypes and three could not be completely characterized. Two of the partially characterized parasites presented a unique 286-m1501 allele, which did not appear in any of the other cases in the area. The last autochthonous case identified in late October also presented a unique haplotype (A10-100-151) (see Figure 3). Finally, the one Evrotas case from 2009 had a unique haplotype (A11-107-191) compared to all of the samples from 2011.

#### Other regions of Greece

In other regions of Greece between July and August (n = 5), three different *P. vivax* haplotypes (A5-121-151, A5-135-176 and C1-276-133) appeared in autochthonous cases from Avlida, Orhomenos and Larisa, respectively, while the *P. vivax* samples from Marathon and Kalivia shared the same haplotype, C3-193-142 (Table 2). The isolates from Athens (n = 5) were diverse, with four isolates expressing unique haplotypes, and the remaining isolate undergoing partial characterization (#-286-142).

## Discussion

Malaria outbreaks in regions that eradicated malaria decades ago have been reported from countries such as Singapore (2009) [29] and Korea (1993) [30]. In the Republic of Korea, malaria was eradicated in the late 1970s, but a single case, which occurred in 1993, resulted in the re-introduction of *P. vivax* malaria in the country in the years that followed [30]. During the past 20 years, few confirmed autochthonous cases of *P. vivax* malaria in the European Union have been reported in Italy (Maremma, 1997) [31], France (Corsica, 2006) [32] and Spain (Aragon, 2010) [33].

In Greece, autochthonous cases have been scarce but have occurred annually since 2009 [34]. The outbreak in 2011, however, was characterized by an unusually higher number of malaria cases, and hence required a study, which was more thorough at the molecular level, in order to expand the epidemiological findings and the related applicability in the region.

The *Pvmsp-3α* gene is one of the most variable polymorphic genes and has been widely used to study the diversity of *P. vivax* and in the discrimination of multiple infections in epidemiological studies [35-38]. The MS m1501 and m3502 have been shown to have high diversity among parasites from Southeast Asia [22,39,40]. Consequently, the combined usage of these three molecular markers is expected to provide a good resolution to discriminate the *P. vivax* strains.

The frequency of the A-genotype among the samples that produced conclusive results for the *Pvmsp-3α* gene was high (0.86) which is in agreement with the high frequency reported from other areas studied [41-43]. The diversity of the MS studied was higher, with 12 different MS m1501 alleles and seven MS m3502 alleles. Higher diversity of the m1501 locus has been reported mainly from Asian populations, while the opposite has been found with higher diversity of m3502 in South American and some Asian populations [26,40].

*Pvmsp-3α* and the MS combined showed multiple *P. vivax* haplotypes expressed in the autochthonous cases in various areas of Greece, which indicates the existence of multiple sources of infection. This is further supported by the haplotype diversity of the imported cases derived from the area of Athens and the unique haplotype of the 2009 sample. In Avlida, Orhomenos and Larisa, only a single autochthonous case was observed, suggesting that the region did not present suitable conditions to sustain continuous transmission (e g, low abundance of mosquito vectors and/or low number of *Plasmodium* carriers), or that the response of local health authorities disrupted the cycle of the parasite.

The two cases derived from East Attica (Marathon and Kalivia) had a common haplotype, indicating possible continuous local transmission, despite the long distance between the locations (approximately 30 km) or common exposure to an imported case. This may be also explained by frequent commuting of inhabitants between these two areas. Although examination of a higher number of molecular markers may increase the resolution of genotypic characterization and eventually separate the common haplotype into two distinct haplotypes, this area should be subject to careful monitoring of malaria transmission in the future. This recommendation is further substantiated by a 2004 entomological survey in the region of Marathon, which identified a high abundance of *An. saccharovi*[19], and recent reports of autochthonous cases in the area [44].

In the municipality of Evrotas, common haplotypes were identified in up to 14 cases. Although this study did not have access to the *P. vivax* genetic material from all infected individuals in Laconia, the data indicate relatively limited diversity and that the spread of only a few *P. vivax* isolates was the main cause of the local outbreak. Additionally, limited autochthonous cases in Evrotas have been reported during the last four years, possibly rendering this an area of local transmission [45,46] culminating in the outbreak in 2011. The equal number of imported and autochthonous cases that share the same genotype indicates that a small number of cases, classified as imported, may have been locally infected. Misclassification was due to lack of documentation and unreliable information of the travel history.

Following the Evrotas outbreak in 2011, the Greek authorities enhanced vector control activities by large-scale spraying with insecticides, health information campaigns, enhanced surveillance and active case finding in affected areas. A possible effect of these activities is that only 20 autochthonous *P. vivax* cases have been identified in four different regions in Greece since then. Of these, ten cases were from Evrotas [46]. In 2013, no autochthonous cases have been reported as at 20 September, 2013.

## Conclusions

The genotypic variability of the majority of the samples studied indicates that a relatively large number of *P. vivax* strains were imported into Greece in various areas of the country, thus increasing the risk of re-introduction of the disease. Continuous transmission in the region of Evrotas and possibly in East Attica indicates that further control measures are urgently needed, especially in these areas. Finally, in cases where medical or travel history cannot be obtained, methods of molecular epidemiology may prove very useful for the correct classification of the cases.

## Competing interests

The authors declare that they have no competing interests.

## Authors’ contributions

GS, MA, NCV, and ICB participated in the design of the study. GS, MLS, EP, NT, and HHH carried out the molecular genetic studies. CH, MT and JK provided most of the blood samples. GS, MA and MLS drafted the manuscript. ICB, NCV, CH, and JK critically revised and gave final approval of the manuscript. All authors read and approved the final manuscript.
